# Application of delayed luminescence measurements for the identification of herbal materials: a step toward rapid quality control

**DOI:** 10.1186/s13020-019-0269-2

**Published:** 2019-10-28

**Authors:** Mengmeng Sun, Shengpeng Wang, Yong Jing, Li Li, Min He, Yusheng Jia, Eduard van Wijk, Yitao Wang, Zhihong Wang, Mei Wang

**Affiliations:** 1 Changchun University of Chinese Medicine, No. 1035, Boshuo Rd, Jingyue Economic Development District, Changchun, 130117 China; 2 Leiden University-European Center for Chinese Medicine and Natural Compounds, Institute of Biology, Leiden University, Sylviusweg 72, Leiden, 2333 BE The Netherlands; 3 SKL of Quality Research in Chinese Medicine, Institute of Chinese Medical Sciences, University of Macau, N22 Avenida da Universidade, Taipa, Macau; 4 College of Pharmacy, Chengdu University of Traditional Chinese Medicine, Chengdu, 611137 China; 5 SU BioMedicine, Postbus 546, 2300AM, Leiden, The Netherlands; 6 Shenzhen Huakai Traditional Chinese Medicine and Natural Medicine Research Center, Shenzhen, 518114 China; 7 Capital Medical University Subsidiary Beijing Hospital of Traditional Chinese Medicine, No. 23, Backstreet of Art Gallery, Dongcheng District, Beijing, 100010 China; 8 Beijing Institute of Chinese Medicine, Shuiche Alley Xinjiekou, No. 13, Xicheng District, Beijing, 100035 China; 9 Meluna Research, Koppelsedijk 1-a, 4191 LC Geldermalsen, The Netherlands

**Keywords:** Delayed luminescence, Herbal materials, Identification, Quality control

## Abstract

**Background:**

Herbal materials are widely used as medicinal products, dietary supplements, food, and spices. With increased consumption, the safety, quality, and efficacy of herbal materials are becoming more relevant. The authenticity of herbal materials plays an important role in herbal quality control, and there is an urgent need to develop a simple, direct, objective, rapid, and inexpensive measurement tool for the identification of herbal materials for the purpose of quality control.

**Methods:**

Delayed luminescence (DL) was used to measure authentic and counterfeit herbal materials. A hyperbolic function was used to extract four properties from the DL curves of the herbal materials. Statistical tools, including Student’s *t* test and Principal Component Analysis, were used to differentiate authentic and counterfeit herbal materials based on the DL properties.

**Results:**

Our results showed that authentic and counterfeit herbal materials could be identified based on the DL properties as follows: (a) authentic versus counterfeit materials; (b) authentic versus adulterated materials; (c) authentic versus sulfur-fumigated materials; as well as (d) authentic versus dyed materials.

**Conclusion:**

The simple, direct, rapid, and inexpensive measurements offered by DL potentially offer a novel technique for the identification of Chinese herbal materials. However, the establishment of a valid database will be the next step toward the possible application of this technique, which would contribute significantly to the development of a novel digital tool for the quality control of herbal materials.

## Background

Herbal medicine has long been used in health and disease treatment in China, and the use of herbal products has increased globally in recent years [1, 2]. In Chinese culture, food and medicine are not clearly separated, and thus a significant amount of herbal materials are used both in medicinal applications and as food supplements (e.g., Ginseng Radix et Rhizoma, etc.) [3]. Herbal materials are thus widely used as additives to foods and spices either during the culinary preparation of meals at home or in the industrial production of processed functional foods and dietary supplements [4]. Therefore, the safety, quality, and efficacy of herbal materials are becoming increasingly important [5]. Chromatography and mass spectrometry are the most common methods and have provided many important information to help the quality assessment on herbal materials [6]. In addition, Raman, mid-infrared and near-infrared spectroscopy as non-destructive and fast measurement tools have been used to discriminated raw and processed herbs [7–9]. Moreover, some novel techniques (e.g. G-quadruplex-based platform) have been used to determine pesticide residues or heavy metals in order to ensure the safety of herbs [10–12].

The authenticity of herbal materials plays an important role in herbal quality control, since the use of counterfeit herbal materials may lead to major health risks [13]. Counterfeit herbs come in various forms, including the replacement of authentic herbal materials with other materials, the adulteration of the authentic products with other substances, and the use of inappropriate processing methods (e.g., sulfur fumigation and dyeing) for fraudulent practices [14, 15]. Therefore, many technologies have been developed to identify counterfeit herbal materials, including morphological and microscopic methods, chromatography and spectrum photometer analyses, molecular biology assays, and biomimetic technologies (e.g., electronic noses and tongue) [16, 17]. However, well-trained and experienced experts are needed to operate these technologies, and most of these analytical methods are time-consuming and expensive. For manufacturing practices, simple and immediately analyzable results are appropriate for on-site digital quality control purposes. There is thus an urgent need to develop a simple, direct, rapid, objective, and inexpensive measurement tool for the identification of herbal materials.

Recently, delayed luminescence (DL) measurements have been used in herbal quality assessments [18–23]. Various dry herbal materials emit long-term and weak luminescence after exposure to illumination. This DL technique has been used to determine the differences and changes in herbal materials with various characters, such as the variation in the growing environment, the processing of the herb, the age of the herb, and in some cases the properties of the herbal materials according to Chinese medicine theory. In addition, DL can be linked to specific chemical components that are indicators of the quality and bioactivity of herbal materials. Therefore, DL has the potential to be developed into a robust new technique for assessing the quality of herbs.

Here, we performed DL measurements in order to evaluate whether DL properties could be used to identify counterfeit herbal materials. A description of the DL dynamics from dry herbal materials is presented, focusing on different forms of counterfeit herbs, including (a) authentic and counterfeit materials; (b) authentic and adulterated materials; (c) authentic and sulfur-fumigated materials; and (d) authentic and dyed materials. The results showed that different DL dynamics exist between authentic and counterfeit herbal materials. Therefore, DL may be further explored for the application in herbal identification and quality control.

## Materials and methods

### Information of experimental design and resources

The information regarding the experimental design, statistics, and resources used in this study are attached in the minimum standards of reporting checklist.

### Herbal materials

The authentic herbal materials and their corresponding nonauthentic herbal materials were purchased in five batches from the Chinese Herbal Pharmacy in Beijing (Beijing, China). These herbal materials are listed in Table 1.

**Table 1:** The authentic herbal materials and their corresponding nonauthentic herbal materials

No	Authentic herbal materials	The corresponding nonauthentic herbal materials	Similarities	References
1	Cyperi rhizoma (*Cyperus rotundus* L.)	Semiaquilegiae radix (*Semiaquilegia adoxoides* (DC.) Makino)	Morphological appearance; color	[24]
2	Atractylodis macrocephalae rhizoma (*Atractylodes macrocephala* Koidz.)	Aucklandiae radix (*Aucklandia lappa* Decne.)	Morphological appearance; characteristics	[25]
3	Pulsatillae radix (*Pulsatilla chinensis* (Bge.) Regel)	Rhapontici radix (*Rhaponticum uniflorum* (L.) DC.)	Morphological appearance; color	[26]
4	Ginseng radix et rhizoma (*Panax ginseng* C. A. Mey.)	Phytolaccae radix (*Phytolacca acinosa* Roxb.)	Morphological appearance; color; Chinese name	[27, 28]
		Glehniae radix (*Glehnia littoralis* Fr. Schmidtex Miq.)		
5	Salviae miltiorrhizae radix et rhizoma (*Salvia Miltiorrhiza* Bge.)	Dipsaci radix (*Dipsacus asper* Wall. ex Henry)	Morphological appearance; color	[29, 30]
6	Peucedani radix (*Peucedanum praeruptorum* Dunn)	Saposhnikoviae radix (*Saposhnikovia divaricata* (Turcz.) Schischk.)	Morphological appearance; characteristics	[31]

Angelicae sinensis radix (*Angelica sinensis* (Oliv.) Diels), Dioscoreae rhizoma (*Dioscorea opposita* Thunb.), Gastrodiae rhizoma (*Gastrodia elata* Bl.), Angelicae dahuricae radix (*Angelica dahurica* (Fisch. ex Hoffm.) Benth. et Hook. f.), Codonopsis radix (*Codonopsis pilosula* (Franch.) Nannf.), and their sulfur-fumigated samples were supplied by the Chengdu University of Traditional Chinese Medicine, as well as Cinnabaris, Croci stigma (*Crocus sativus* L.), and their dyed samples. Rhei radix et rhizoma (Rhubarb; *Rheum palmatum* L. and *Rheum tanguticum* Maxim. ex Balf.) samples were collected at various elevations in western China (Additional file 1: Table S1). These rhubarb samples were obtained as gifted samples from the Beijing Institute of Chinese Medicine. All herbal samples were verified by Dr. Mei Wang and Mr. Yusheng Jia, and these herbal materials were deposited at Leiden University, Leiden, Netherlands.

### Delayed luminescence

#### Sample preparation

Each herbal sample was crushed using a model QE-100 grinder (Yili Company, Zhejiang Province, China) and passed through a standard sieve to obtain 150-μm particles. All the herbal materials were kept in a dark, light-tight box containing 35-mm silica gel (Boom BV, Meppel, Netherlands) at room temperature for 16 h before DL measurements were conducted [20].

#### DL measurements

The DL measurement procedure was described previously [20]. The equipment used to measure DL was provided by Meluna Research (Geldermalsen, Netherlands) and involved a type 9558QB photomultiplier tube (Electron Tubes Enterprises Ltd., Ruislip, UK) positioned vertically on a dark chamber kept at 22 °C. The photomultiplier tube has a cathode end (51 mm diameter) with a sensitive range at 300–800 nm. The photomultiplier tube was cooled to − 25 °C to decrease the dark count rate to 10 counts per second. The DL signal was amplified utilizing a fast preamplifier (Type 9301, ORTEC, Oak Ridge, TN). Data were acquired using a personal computer containing a model 6602 counting card (National Instruments, Austin, TX).

Each herbal material was used to prepare 1-g samples. Each 1-g sample was placed in a Petri dish (35-mm diameter) and exposed to a white halogen excitation source (Model 284-2812, Philips, Germany) for 10 s. For each 1-g sample, the DL signal was measured three consecutive times. The total number obtained from all three measurements in each sample was used to analyze the DL properties of that particular herb. DL kinetics were obtained by recording the number of counts in consecutive 0.05-s periods for a total of 60 s, resulting in a total of 1200 data points.

#### Data processing and statistical analysis

The DL decay curve of each sample, measured over a 60-s period, was fit to the following hyperbolic function [22]:

$$ I_{\left( t \right)} = \frac{{I_{0} }}{{\left( {1 + \frac{t}{Tau}} \right)^{Beta} }} $$

$$ T = \left( {e^{{\frac{1}{ Beta}}} - 1} \right) \times Tau $$

where T is the decay time of the DL curve, Beta is an index factor associated with the rate of DL decay, and Tau and I_0_ represent the DL characteristics and initial intensity, respectively. The properties of the three measurements were averaged and used to represent the DL properties of each herbal sample. Principal component analysis (PCA) and orthogonal projections to latent structures-discriminant analysis (OPLS-DA) were used to indicate the level of discrimination between the DL properties of the different herbal samples using tools provided in the MetaboAnalyst software package (http://www.metaboanalyst.ca). A two-tailed, unpaired Student’s *t* test was used (SPSS version 23.0) to compare the DL properties between different herbal samples. Differences were considered significant at *p *< 0.05.

### Sample preparation and high-performance liquid chromatography (HPLC) analysis for the rhubarb materials

In order to determine the contents of 14 bioactive compounds in the rhubarb samples, HPLC analysis was performed using an Agilent 1100 system (Agilent Technologies, Palo Alto, CA). The sample preparation, HPLC analysis methods, and statistical methods have been reported previously [19]. The reference compounds (aloe-emodin, rhein, emodin, chrysophanol, physcion, gallic acid, (+)-catechin, sennoside A, and sennoside B) were purchased from the National Institute for Food and Drug Control (Beijing, China). The purity of all reference compounds was > 98%. A two-tailed, unpaired Student’s *t* test was used (SPSS version 23.0, Armonk, NY, USA) to compare the contents of the detected bioactive compounds between two rhubarb species. Differences were considered significant at *p *< 0.05.

## Results

### DL properties of rhubarb samples between different subspecies

**Fig. 1:**
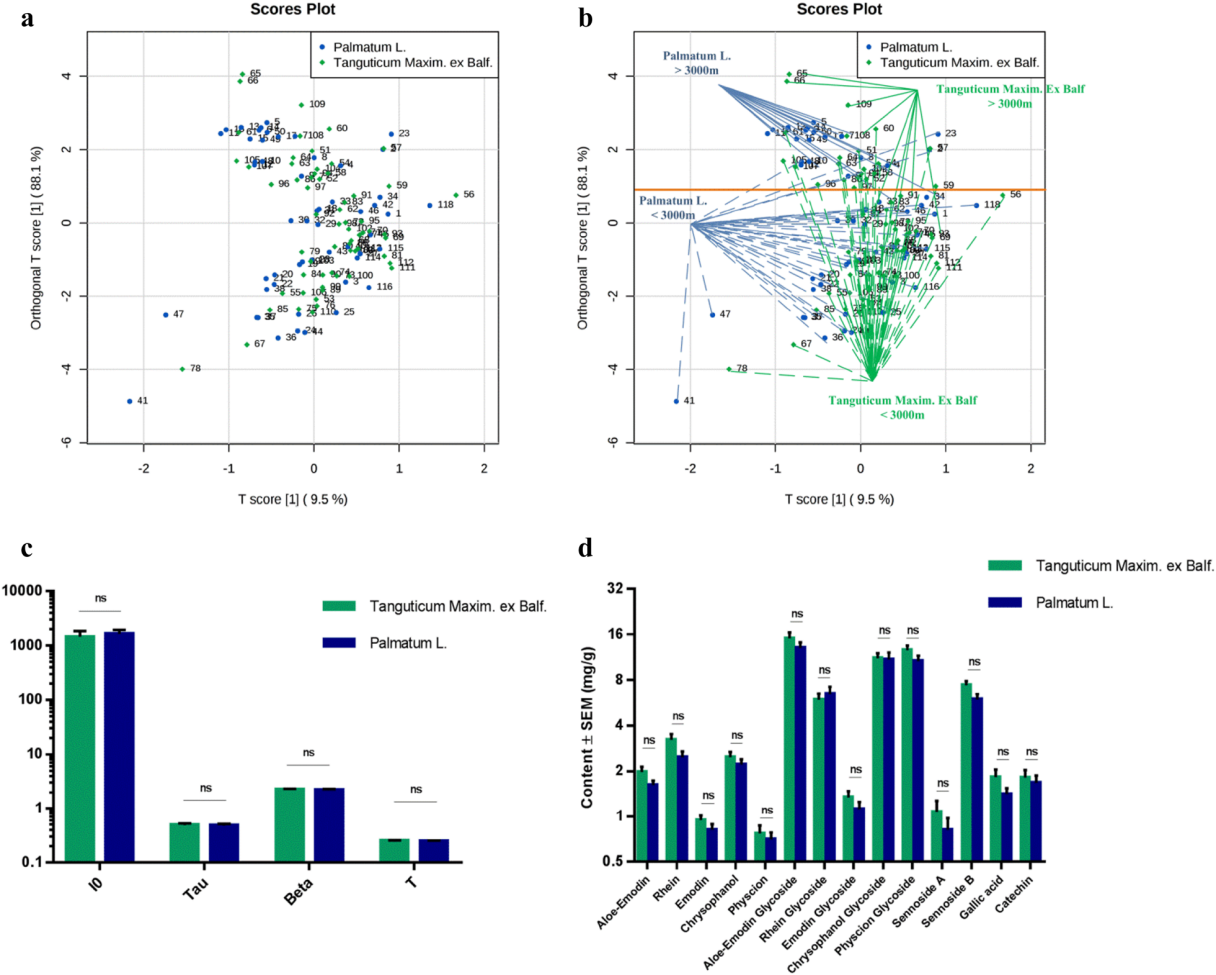
The features of DL properties and the contents of chemical components between two rhubarb subspecies. **a** OPLS-DA score plots of the DL properties obtained from all batches of two rhubarb subspecies; **b** Rhubarb samples grown over (solid lines) or under (dash lines) 3000 m were marked on the OPLS-DA score plots, the orange horizontal line indicates the separation between *Rheum palmatum* L. samples grown over or under 3000 m; **c** Histograms comparing the DL properties between two rhubarb subspecies. Mean ± SD, *ns* no significant difference; **d** Histograms comparing the bioactive components between two rhubarb subspecies. Mean ± SEM, *ns* no significant difference

Rhubarb samples (55 batches of *R. palmatum* and 63 batches of *R. tanguticum*) were submitted to DL and HPLC analysis. Each rhubarb sample was measured for DL three consecutive times, and the data were pooled in order to determine the DL characteristics of the rhubarb samples. Next, supervised OPLS-DA was used to investigate the variance in the four DL properties of the rhubarb samples. The results showed that there was no clear separation between the two rhubarb subspecies (Fig. 1a), and there was no significant difference between the specific DL properties (Fig. 1c). These rhubarb samples were collected from different altitudes at a threshold of 3000 m (Additional file 1: Table S1). A previous study showed that the DL properties of *R. palmatum* samples were sensitive to altitude [19]. We thus labeled the rhubarb samples grown at an altitude above or below 3000 m on the OPLS-DA plot (Fig. 1b). It is evident that the *R. palmatum* samples were reasonably separated at an altitude of 3000 m, with only a few misclassified samples, which is consistent with previous results [19]. This separation has been marked using an orange horizontal line on Fig. 1b. However, the *R. tanguticum* samples could not be separated based on the same altitude threshold, and many samples were misclassified according to the 3000 m threshold (Fig. 1b). Since different luminescence properties reflect the different chemical diversities of molecules [32, 33], the bioactive compound contents of these rhubarb samples were also measured. No significant difference was detected between the two rhubarb subspecies (Fig. 1d).

### The DL technique can discriminate between authentic and nonauthentic herbal materials

**Fig. 2:**
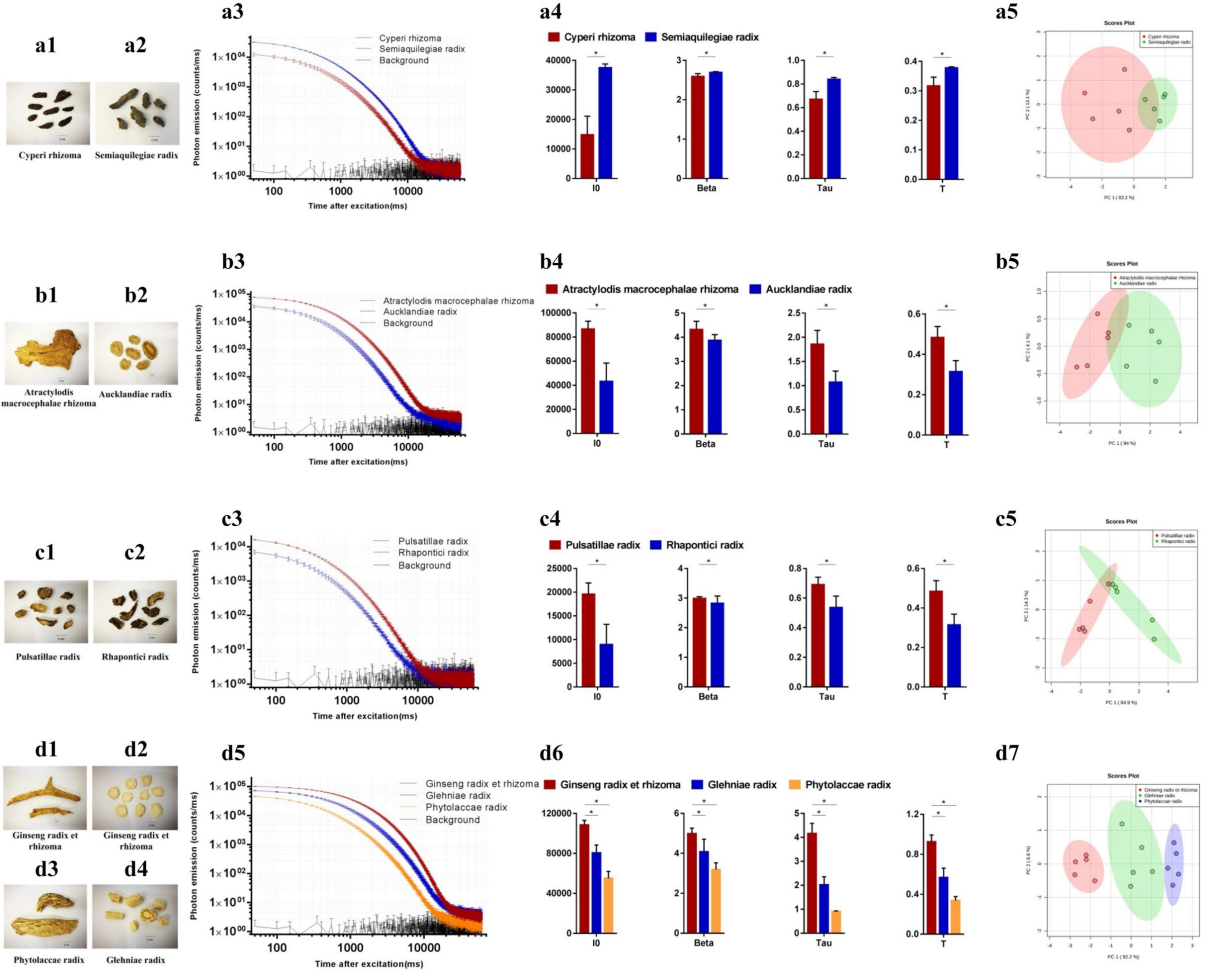
The differences of DL properties between authentic and nonauthentic herbal materials. **a1** Cyperi rhizoma (fragmented materials); **a2** Semiaquilegiae radix (fragmented materials); **a3** DL decay curves. Data are plotted as the mean ± SEM. Note that the data are plotted on a log–log scale; **a4** Histograms comparing the DL properties between Cyperi rhizoma and Semiaquilegiae radix. Mean ± SD, *p < 0.05; **a5** PCA score plots of the DL properties obtained from all batches of Cyperi rhizoma (Red) and Semiaquilegiae radix (Green); **b1** Atractylodis macrocephalae rhizoma (sliced materials); **b2** Aucklandiae radix (sliced materials); **b3** DL decay curves. Data are plotted as the mean ± SEM. Note that the data are plotted on a log–log scale; **b4** Histograms comparing the DL properties between Atractylodis macrocephalae rhizoma and Aucklandiae radix. Mean ± SD, *p < 0.05; b5) PCA score plots of the DL properties obtained from all batches of Atractylodis macrocephalae rhizoma (Red) and Aucklandiae radix (Green); **c1** Pulsatillae radix (fragmented materials); **c2** Rhapontici radix (fragmented materials); **c3** DL decay curves. Data are plotted as the mean ± SEM. Note that the data are plotted on a log–log scale; **c4** Histograms comparing the DL properties between Pulsatillae radix and Rhapontici radix. Mean ± SD, *p < 0.05; **c5** PCA score plots of the DL properties obtained from all batches of Pulsatillae radix (Red) and Rhapontici radix (Green); **d1** Ginseng radix et rhizoma (fragmented materials); **d2** Ginseng radix et rhizoma (sliced materials); **d3** Phytolaccae radix (fragmented materials); **d4** Glehniae radix (sliced materials); **d5** DL decay curves. Data are plotted as the mean ± SEM. Note that the data are plotted on a log–log scale; **d6** Histograms comparing the DL properties among three materials. Mean ± SD, *p < 0.05; **d7** PCA score plots of the DL properties obtained from all batches of Ginseng radix et rhizoma (Red), Glehniae radix (Green) and Phytolaccae radix (Purple)

Nonauthentic or counterfeit herbal materials refer to herbs that possess similarities in morphological appearance, characteristics, or Chinese name compared to that of the authentic herbal materials but may be cheaper in price or mistakenly purchased. These herbs possess different chemical compositions and therefore reflect different bioactivities [34]. We selected several common nonauthentic herbal materials and their corresponding authentic herbs (Table 1), which had been reported in the literature, for our DL study [24–28]. Each herbal material was prepared in five independent batches, and each batch was measured for DL three consecutive times. Thereafter, all data in the 15 DL measurements were pooled in order to indicate the DL characteristics of the specific herb for statistical purposes. The statistical results are shown in Fig. 2. The results illustrate that the nonauthentic herbal materials exhibit different DL kinetics (Fig. 1a3, b3, c3, and d5) and significantly different DL properties (i.e., I0, Beta, Tau and T; Fig. 1a4, b4, c4, and d6) compared to the authentic materials. In addition, the PCA score plots revealed different clusters between the authentic and nonauthentic herbal materials (Fig. 1a5, b5, c5, and d7).

### The DL technique is able to detect differences between authentic and adulterated herbal materials

**Fig. 3:**
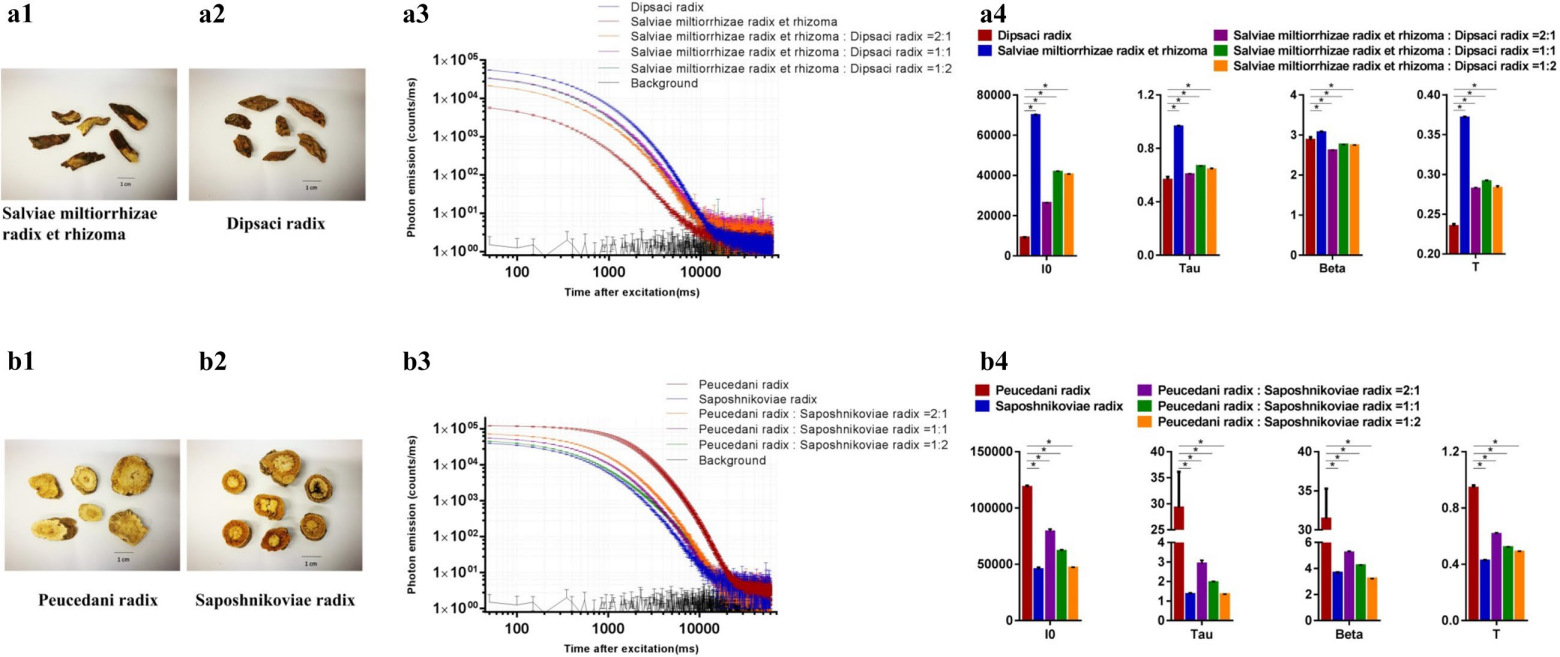
The differences of DL properties between authentic and adulterated herbal materials. **a1** Salviae miltiorrhizae radix et rhizoma (fragmented materials); **a2** Dipsaci radix (fragmented materials); **a3** DL decay curves. Data are plotted as the mean ± SEM. Note that the data are plotted on a log–log scale; **a4** Histograms comparing the DL properties among samples. Mean ± SD, *p < 0.05; **b1** Peucedani radix (sliced materials); **b2** Saposhnikoviae radix (sliced materials); **b3** DL decay curves. Data are plotted as the mean ± SEM. Note that the data are plotted on a log–log scale; **b4** Histograms comparing the DL properties among samples. Mean ± SD, *p < 0.05

Adulterated herbal material refers to the mixing of nonauthentic herbal materials into authentic materials. It is difficult to distinguish adulterated herbal materials by the naked eye, particularly in the powder form. Here, Salviae miltiorrhizae radix et rhizoma (authentic materials) and Dipsaci radix (nonauthentic materials) [29, 30], Peucedani radix (authentic materials) and Saposhnikoviae radix (nonauthentic materials) [31], and their adulterated samples (Table 1) were used to measure DL. Figure 3a3 and b3 show the significantly different DL decay curves of the authentic materials compared to the adulterated materials in three different ratios. In addition, the DL decay curves of the adulterated materials were situated between the authentic and nonauthentic herbal materials. As the proportion of nonauthentic materials decreased, the DL decay curves of the adulterated materials become closer to that of the authentic materials but still demonstrated significant differences in specific DL properties (Fig. 3a4 and b4).

### Herbal materials can be separated by DL measurement with or without sulfur fumigation

**Fig. 4:**
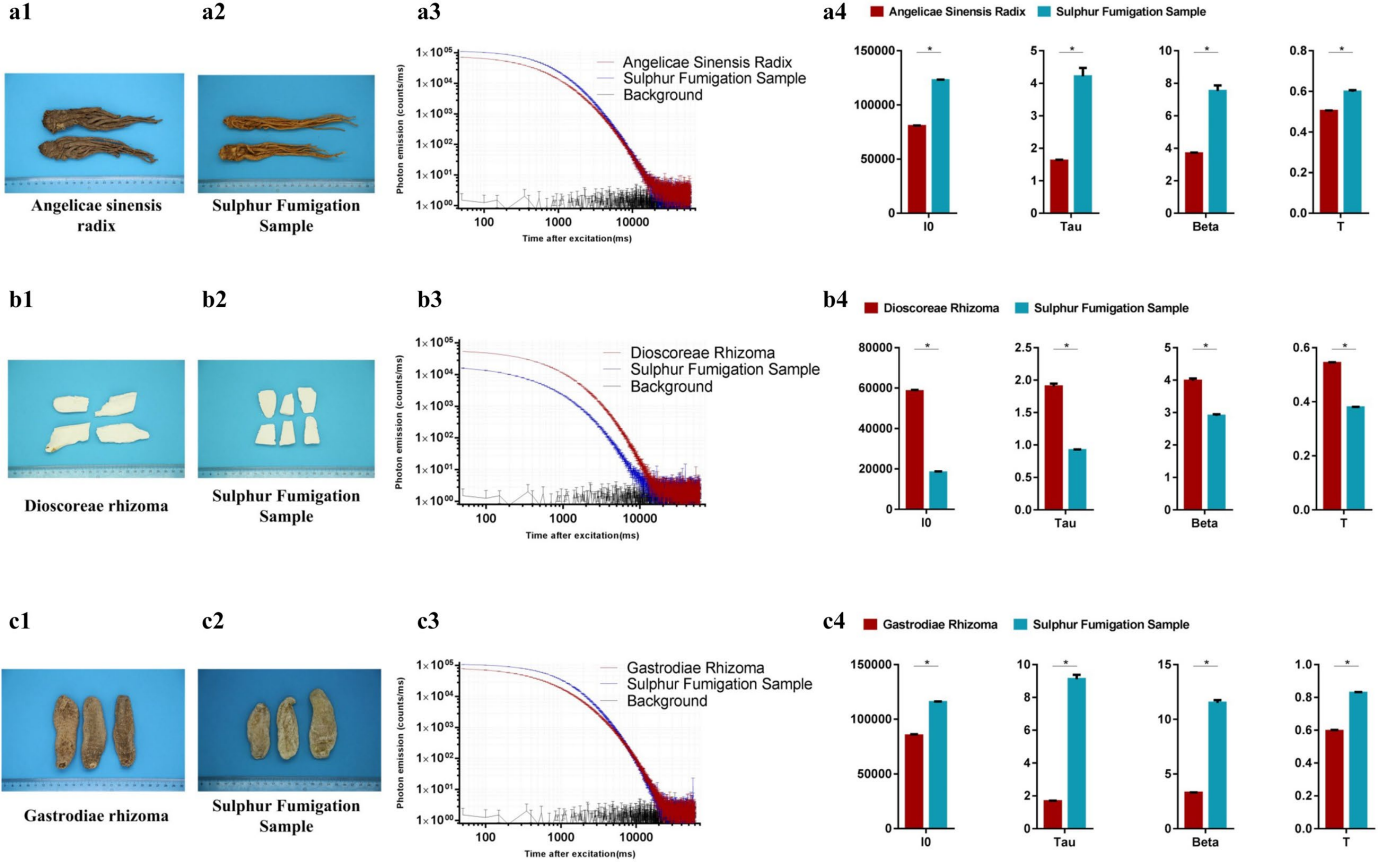
The differences of DL properties between authentic and sulfur fumigation herbal materials. **a1** Angelicae sinensis radix (whole materials); **a2** Angelicae sinensis radix (sulphur fumigation sample, whole materials); **a3** DL decay curves. Data are plotted as the mean ± SEM. Note that the data are plotted on a log–log scale; **a4** Histograms comparing the DL properties between Angelicae sinensis radix and its sulphur fumigation sample. Mean ± SD, *p < 0.05; **b1** Dioscoreae rhizoma (sliced materials); **b2** Dioscoreae rhizoma (sulphur fumigation sample, sliced materials); **b3** DL decay curves. Data are plotted as the mean ± SEM. Note that the data are plotted on a log–log scale; **b4** Histograms comparing the DL properties between Dioscoreae rhizoma and its sulphur fumigation sample. Mean ± SD, *p < 0.05; **c1** Gastrodiae rhizoma (whole materials); **c2** Gastrodiae rhizoma (sulphur fumigation sample, whole materials); **c3** DL decay curves. Data are plotted as the mean ± SEM. Note that the data are plotted on a log–log scale; **c4** Histograms comparing the DL properties between Gastrodiae rhizoma and its sulphur fumigation sample. Mean ± SD, *p < 0.05

Sulfur fumigation is a processing method used for herbal materials in China in order to prolong the storage life, improve the appearance of herbs, remove borers, and prevent mildew [35]. However, modern studies have shown that sulfur fumigation may pollute the herbal materials with sulfur dioxide and heavy metals [35]. In addition, the bioactive components of the herbal materials may be altered by sulfur fumigation [35]. Therefore, the sulfur fumigation method was banned by the Chinese Food and Drug Administration in 2010. However, sulfur-fumigated herbal materials still exist in the market at present [35]. Here, we compared Angelicae sinensis radix, Dioscoreae rhizoma, Gastrodiae rhizoma, and their sulphur-fumigated samples using DL measurements. The results show that the DL decay kinetics and four DL properties of the herbal samples fumigated by sulfur changed significantly compared to their raw materials (Fig. 4). Moreover, Angelicae dahuricae radix, Codonopsis radix, and the corresponding sulfur-fumigated samples also demonstrated different DL kinetics (Additional file 2: Fig. S1).

### Dyed herbal materials can be identified by DL measurements

**Fig. 5:**
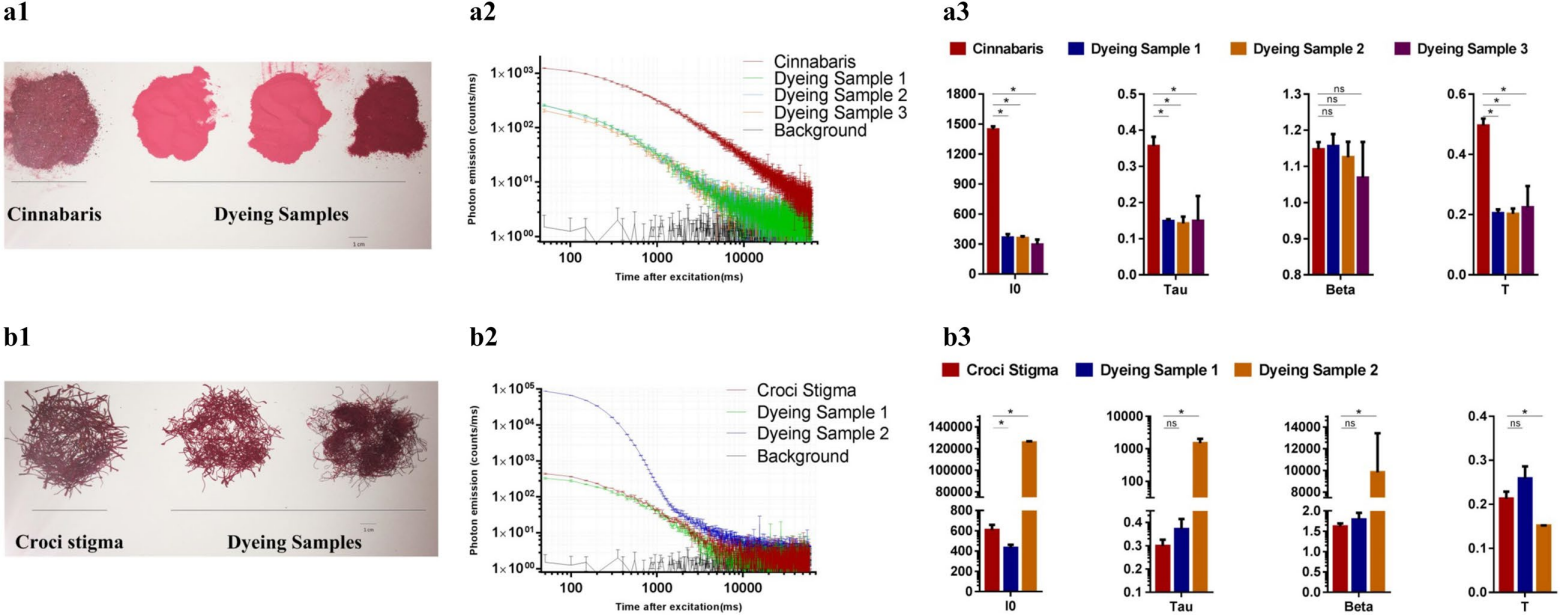
The differences of DL properties between authentic and dyeing herbal materials. **a1** Cinnabaris (powder materials) and dyeing samples (unknown powder materials); **a2** DL decay curves. Data are plotted as the mean ± SEM. Note that the data are plotted on a log–log scale; **a3** Histograms comparing the DL properties between cinnabaris and its dyeing samples. Mean ± SD, *p < 0.05, ns, no significant difference; **b1** Croci stigma (stigma materials) and dyeing samples (unknown stigma materials); **b2** DL decay curves. Data are plotted as the mean ± SEM. Note that the data are plotted on a log–log scale; **b3** Histograms comparing the DL properties between Croci stigma and its dyeing samples. Mean ± SD, *p < 0.05, ns, no significant difference

Dyed herbal materials are common counterfeit products of cinnabar (mineral materials) and Croci stigma in the market [36, 37]. To identify dyed herbal materials, DL was used to measure authentic materials of cinnabar and Croci stigma and their corresponding dyed materials. The results showed that the DL kinetics and the majority of the DL properties of authentic cinnabar differed significantly compared to the dyed samples (Fig. 5a1–a3). For Croci stigma materials, dyed sample 2 demonstrated significant differences in DL properties compared to the authentic materials. However, dyed sample 1 displayed similar DL decay curves as the authentic materials, and only DL property I_0_ differed significantly from the authentic materials (Fig. 5b1–b3). These results indicate that the identification of dyed samples using DL measurements should be further investigated.

## Discussion

Two subspecies of rhubarb, *Rheum palmatum* L. and *Rheum tanguticum* Maxim. ex Balf., are listed in the Chinese Pharmacopeia [38], indicating that there is no significant difference in the application of these two subspecies for therapeutic purposes according to the principle for Chinese medicine theory. As herb’s therapeutic effects mainly depend on bioactive components, we measured the contents of fourteen bioactive compounds of rhubarb samples. There was no significant difference between the two rhubarb subspecies. The results may explain why the Chinese pharmacopeia does not distinguish between different rhubarb sub-species. Next, we were interested in whether DL was able to separate two subspecies of rhubarb with the same therapeutic applications, and the statistical data indicated that there was no reasonable separation between different rhubarb subspecies. In addition, the DL properties of *R. palmatum* samples were sensitive with environmental factors, which is consistent with previous results, but *R. tanguticum* samples were not applicable. These results suggest that when the observation scope is extended to more than one rhubarb subspecies, the influence of environmental factors on DL may weaken. The results emphasize the relationship between chemical composition and DL properties. When there are differences in the chemical profiles between herbal materials—which can be caused by environmental factors [19, 39, 40]—DL might reflect these differences. The DL characteristics of herbal materials are closely related to their chemical constituents. Due to this finding, we wondered whether DL technology could be applied to distinguish counterfeit or adulterated herb materials, as these certainly have different chemical profiles.

Many counterfeit herbs do not belong to the same species or genus as the authentic herbs [24–28], and thus they typically possess different bioactive components and quality indicators [38, 41–49]. Therefore, it is worth investigating whether there is any difference in DL properties between the authentic and counterfeit/adulterated herbs as a result of their different chemical backgrounds. Our results indicate that the DL measurements may be sensitive enough to distinguish nonauthentic herbal materials, and the variations in DL properties between the authentic and nonauthentic herbal materials may be mainly as a result of their different chemical profiles [41–49]. In particular, the chemical components of the herbal materials may be altered by sulfur fumigation [35]. Our previous study indicated that herbal materials with higher polysaccharide contents usually emit stronger luminescence [18]. The polysaccharide contents of Angelicae sinensis radix can be increased using sulfur fumigation [50, 51]. This may be the reason for the higher intensity of the DL decay curves and I_0_ of the sulfur-fumigated sample of Angelicae sinensis radix (Fig. 4a3 and a4). Conversely, the fumigation of Dioscoreae rhizoma with a large amount of sulfur can significantly decrease polysaccharides [52]. The sulfur-fumigated sample of Dioscoreae rhizoma thus demonstrates a much lower photon emission intensity compared to its raw material (Fig. 4b3 and b4). In addition, herbal dry extracts with fewer glycosides may exhibit increased DL strength [21]. The main bioactive ingredients of Gastrodiae rhizoma are glycosides, such as gastrodin, parishin, parishin B, parishin C, and parishin E [53]. These bioactive ingredients can be decreased during sulfur fumigation [54, 55]. The sulfur-fumigated sample of Gastrodiae rhizoma thus exhibited stronger DL intensity (Fig. 4c3 and c4).

Delayed luminescence is a photo-induced ultra-weak photon emission [56]. The properties of weak photon emission are influenced by molecular structures and interactions [32], in particular the long chain molecules [57, 58]. Polysaccharides are widespread long-chain macromolecules commonly found in herbal materials. The structure of polysaccharides is polymeric carbohydrate molecules composed of long chains of monosaccharide units bound together by glycosides linkages. When all the monosaccharide units in a polysaccharide are the same type, the polysaccharide is called a homo-polysaccharide, such as amylose and cellulose. When more than one type of monosaccharide is present they are called hetero-polysaccharides (e.g. Hyaluronic acid). Grasso et al. reported significantly different DL kinetics of intensities and decay time intervals between amylose and cellulose, which share the same glucose-based repeat units but have differently orientated structures [59]. And the DL lasts in the large interval of time, which indicates existence of the excited electron states with large lifetimes, such as excitons and/or solitons [59]. This finding has important implications in studying the DL biophysical mechanism of polysaccharides. As our research shows that the polysaccharides and glycosides of herbal materials are related to the herbal DL properties. The soliton mechanism of DL [60, 61] may be applicable to understand herbal DL kinetics. However, the investigation of DL properties of polysaccharides must be further studied in order to understand the effects to herbal DL characteristics. To the present research, DL may be a sensitive indicator for reflecting the differences in chemical profiles between authentic and counterfeit herbal materials.

## Conclusions

The data presented in this paper show that DL may be a promising tool in the identification of herbal material, as DL properties are sensitive to the variations in the chemical components of the herbal materials. DL may be a potential rapid measurement tool for assisting conventional identification methods, such as morphological and microscopical identification, DNA-barcoding [62], and chromatographic analysis. For the further development of this analytic tool in practice, further data accumulation and confirmation are required. This proof-of-concept study should be further validated using a wider range of herbal samples and a comprehensive database in combination with artificial intelligence to establish an applicable tool for the identification of authentic and counterfeit herbal materials. In addition, the digital assessment of herbal materials has become an inevitable direction in herbal quality control [63]. As a simple, direct, rapid, and inexpensive measurement tool, DL has the potential to be further developed into a digital monitoring tool for herbal materials. Therefore, DL as a novel technique has great potential to contribute to the identification of herbal materials.

## Supplementary information

**Additional file 1: Table S1.** The DL properties and altitudes of rhubarb samples.

**Additional file 2: Figure S1.** The differences of DL properties between authentic and sulfur fumigation herbal materials. A1) Angelicae dahuricae radix (sliced materials); A2) Angelicae dahuricae radix (sulphur fumigation sample, sliced materials); A3) DL decay curves. Data are plotted as the mean ± SEM. Note that the data are plotted on a log-log scale; A4) Histograms comparing the DL properties between Angelicae dahuricae radix and its sulphur fumigation sample. Mean ± SD, *, *p* < 0.05; B1) Codonopsis radix (whole materials); B2) Codonopsis radix (sulphur fumigation sample, whole materials); B3) DL decay curves. Data are plotted as the mean ± SEM. Note that the data are plotted on a log-log scale; B4) Histograms comparing the DL properties between Codonopsis radix and its sulphur fumigation sample. Mean ± SD, *, *p* < 0.05, ns, no significant difference.

## Data Availability

The datasets used in this study are available from the corresponding author upon reasonable request.

## Abbreviations

DL: delayed luminescence
PCA: principal component analysis
OPLS-DA: orthogonal projections to latent structures discriminant analysis
HPLC: high performance liquid chromatography
