# Supplementary material for: Application of delayed luminescence measurements for the identification of herbal materials: a step toward rapid quality control
Source: Chin Med. 2019 Oct 28;14:47. doi: 10.1186/s13020-019-0269-2 (PMC6819577; doi:10.1186/s13020-019-0269-2)
Supplement: Supplementary file 1 — Additional file 1: Table S1. The DL properties and altitudes of rhubarb samples. [file 13020_2019_269_MOESM1_ESM.docx]

| No | Group | I0 | Tau | Beta | T | Sample |  |  |
| --- | --- | --- | --- | --- | --- | --- | --- | --- |
| 16 | <3000m | 7751.116 | 0.767875 | 3.053604 | 0.29725 | Palmatum L. | |  |
| 11 | <3000m | 7544.781 | 0.728236 | 3.00503 | 0.28742 | Palmatum L. | |  |
| 13 | <3000m | 6816.228 | 0.828611 | 3.178587 | 0.306227 | Palmatum L. | |  |
| 5 | <3000m | 6464.339 | 0.901391 | 3.012404 | 0.354915 | Palmatum L. | |  |
| 14 | <3000m | 6116.704 | 0.849194 | 2.983136 | 0.338157 | Palmatum L. | |  |
| 6 | <3000m | 6074.701 | 0.816984 | 2.893324 | 0.337257 | Palmatum L. | |  |
| 15 | <3000m | 5210.83 | 0.750791 | 2.960553 | 0.301524 | Palmatum L. | |  |
| 17 | <3000m | 3918.199 | 0.849496 | 3.169278 | 0.315139 | Palmatum L. | |  |
| 7 | <3000m | 3710.741 | 0.865923 | 2.955127 | 0.348559 | Palmatum L. | |  |
| 12 | <3000m | 3042.059 | 0.59235 | 2.682986 | 0.267505 | Palmatum L. | |  |
| 10 | <3000m | 2748.202 | 0.649252 | 2.945217 | 0.261802 | Palmatum L. | |  |
| 8 | <3000m | 1708.365 | 0.796788 | 3.200331 | 0.292031 | Palmatum L. | |  |
| 9 | <3000m | 1571.21 | 0.608453 | 2.615506 | 0.283192 | Palmatum L. | |  |
| 4 | <3000m | 1391.66 | 0.742539 | 2.664823 | 0.338079 | Palmatum L. | |  |
| 33 | <3000m | 692.1403 | 0.527051 | 2.468733 | 0.261931 | Palmatum L. | |  |
| 34 | <3000m | 463.6108 | 0.639799 | 2.644701 | 0.293945 | Palmatum L. | |  |
| 65 | <3000m | 19188.15 | 1.363675 | 3.800559 | 0.410434 | Tanguticum Maxim. ex Balf. | | |
| 66 | <3000m | 16232.67 | 1.292214 | 3.87482 | 0.380395 | Tanguticum Maxim. ex Balf. | | |
| 109 | <3000m | 5478.182 | 1.253235 | 3.785509 | 0.378613 | Tanguticum Maxim. ex Balf. | | |
| 108 | <3000m | 2827.664 | 0.958921 | 3.583589 | 0.306512 | Tanguticum Maxim. ex Balf. | | |
| 92 | <3000m | 630.57 | 0.446159 | 2.355079 | 0.23563 | Tanguticum Maxim. ex Balf. | | |
| 91 | <3000m | 570.4714 | 0.618476 | 2.776359 | 0.266522 | Tanguticum Maxim. ex Balf. | | |
| 93 | <3000m | 217.2493 | 0.459008 | 2.306338 | 0.244973 | Tanguticum Maxim. ex Balf. | | |
| 90 | <3000m | 216.3943 | 0.237666 | 1.650537 | 0.193778 | Tanguticum Maxim. ex Balf. | | |
| 89 | <3000m | 176.6188 | 0.195366 | 1.448963 | 0.189849 | Tanguticum Maxim. ex Balf. | | |
| 50 | >3000m | 5249.82 | 0.827344 | 2.948765 | 0.333907 | Palmatum L. | |  |
| 49 | >3000m | 4449.227 | 0.775017 | 3.00727 | 0.305671 | Palmatum L. | |  |
| 48 | >3000m | 3001.568 | 0.628357 | 2.874473 | 0.260416 | Palmatum L. | |  |
| 23 | >3000m | 1245.044 | 1.262861 | 3.717281 | 0.388201 | Palmatum L. | |  |
| 2 | >3000m | 1037.191 | 1.060098 | 3.425491 | 0.357535 | Palmatum L. | |  |
| 30 | >3000m | 742.2617 | 0.385592 | 2.19203 | 0.220276 | Palmatum L. | |  |
| 31 | >3000m | 655.5809 | 0.467291 | 2.432291 | 0.236932 | Palmatum L. | |  |
| 32 | >3000m | 619.8844 | 0.407817 | 2.252172 | 0.224964 | Palmatum L. | |  |
| 18 | >3000m | 588.0426 | 0.50362 | 2.701407 | 0.222408 | Palmatum L. | |  |
| 29 | >3000m | 530.5119 | 0.402977 | 2.198634 | 0.22978 | Palmatum L. | |  |
| 42 | >3000m | 446.9764 | 0.569622 | 2.420787 | 0.290479 | Palmatum L. | |  |
| 46 | >3000m | 419.464 | 0.534285 | 2.503876 | 0.25873 | Palmatum L. | |  |
| 47 | >3000m | 414.414 | 0.102359 | 1.4239 | 0.104165 | Palmatum L. | |  |
| 39 | >3000m | 327.0034 | 0.2569 | 1.770873 | 0.193351 | Palmatum L. | |  |
| 21 | >3000m | 320.6172 | 0.204808 | 1.685391 | 0.157613 | Palmatum L. | |  |
| 19 | >3000m | 319.654 | 0.249331 | 1.748168 | 0.189548 | Palmatum L. | |  |
| 28 | >3000m | 317.6866 | 0.265073 | 1.755764 | 0.202928 | Palmatum L. | |  |
| 40 | >3000m | 311.1197 | 0.32953 | 1.771587 | 0.246453 | Palmatum L. | |  |
| 43 | >3000m | 303.4363 | 0.315564 | 1.85294 | 0.214422 | Palmatum L. | |  |
| 20 | >3000m | 302.5605 | 0.217654 | 1.791726 | 0.159813 | Palmatum L. | |  |
| 1 | >3000m | 277.9327 | 0.58952 | 2.724406 | 0.255702 | Palmatum L. | |  |
| 45 | >3000m | 274.5155 | 0.416701 | 2.091668 | 0.253559 | Palmatum L. | |  |
| 22 | >3000m | 274.3853 | 0.189616 | 1.626403 | 0.160285 | Palmatum L. | |  |
| 38 | >3000m | 264.324 | 0.186254 | 1.584047 | 0.150816 | Palmatum L. | |  |
| 118 | >3000m | 262.6975 | 0.670788 | 2.486056 | 0.329993 | Palmatum L. | |  |
| 117 | >3000m | 242.4654 | 0.341105 | 1.908002 | 0.234447 | Palmatum L. | |  |
| 27 | >3000m | 240.6403 | 0.316036 | 1.73909 | 0.244688 | Palmatum L. | |  |
| 114 | >3000m | 214.5446 | 0.311838 | 1.796124 | 0.228412 | Palmatum L. | |  |
| 115 | >3000m | 208.4312 | 0.362525 | 1.856031 | 0.254186 | Palmatum L. | |  |
| 35 | >3000m | 202.5161 | 0.121377 | 1.244901 | 0.144428 | Palmatum L. | |  |
| 37 | >3000m | 197.5558 | 0.125059 | 1.245918 | 0.143339 | Palmatum L. | |  |
| 3 | >3000m | 185.5201 | 0.213595 | 1.387046 | 0.224821 | Palmatum L. | |  |
| 26 | >3000m | 163.5306 | 0.130127 | 1.166736 | 0.176662 | Palmatum L. | |  |
| 41 | >3000m | 152.3323 | 0.031185 | 0.805097 | 0.076803 | Palmatum L. | |  |
| 25 | >3000m | 133.3223 | 0.139153 | 1.07981 | 0.210868 | Palmatum L. | |  |
| 24 | >3000m | 130.4865 | 0.10494 | 1.022533 | 0.171494 | Palmatum L. | |  |
| 36 | >3000m | 127.6775 | 0.096242 | 1.048587 | 0.150451 | Palmatum L. | |  |
| 116 | >3000m | 127.2576 | 0.223729 | 1.423927 | 0.22359 | Palmatum L. | |  |
| 44 | >3000m | 117.3156 | 0.105439 | 1.030451 | 0.17212 | Palmatum L. | |  |
| 61 | >3000m | 6382.822 | 0.780933 | 3.08874 | 0.298264 | Tanguticum Maxim. ex Balf. | | |
| 105 | >3000m | 3890.013 | 0.584464 | 2.704565 | 0.261351 | Tanguticum Maxim. ex Balf. | | |
| 107 | >3000m | 2913.455 | 0.585242 | 2.795901 | 0.251503 | Tanguticum Maxim. ex Balf. | | |
| 60 | >3000m | 2686.046 | 1.077529 | 3.465056 | 0.36046 | Tanguticum Maxim. ex Balf. | | |
| 64 | >3000m | 2302.078 | 0.723178 | 2.920026 | 0.29521 | Tanguticum Maxim. ex Balf. | | |
| 63 | >3000m | 2189.086 | 0.667634 | 2.733452 | 0.294766 | Tanguticum Maxim. ex Balf. | | |
| 51 | >3000m | 2119.754 | 0.826861 | 3.109397 | 0.313091 | Tanguticum Maxim. ex Balf. | | |
| 96 | >3000m | 1846.943 | 0.508299 | 2.438621 | 0.25747 | Tanguticum Maxim. ex Balf. | | |
| 86 | >3000m | 1499.903 | 0.585964 | 2.605772 | 0.271931 | Tanguticum Maxim. ex Balf. | | |
| 77 | >3000m | 1425.452 | 0.614311 | 2.569281 | 0.291719 | Tanguticum Maxim. ex Balf. | | |
| 54 | >3000m | 1344.26 | 0.781266 | 3.043871 | 0.302894 | Tanguticum Maxim. ex Balf. | | |
| 94 | >3000m | 1333.396 | 0.668748 | 2.852776 | 0.280445 | Tanguticum Maxim. ex Balf. | | |
| 104 | >3000m | 1323.663 | 0.725619 | 3.088709 | 0.275919 | Tanguticum Maxim. ex Balf. | | |
| 58 | >3000m | 1197.156 | 0.69493 | 2.852535 | 0.291676 | Tanguticum Maxim. ex Balf. | | |
| 52 | >3000m | 1194.804 | 0.634294 | 2.706723 | 0.283239 | Tanguticum Maxim. ex Balf. | | |
| 97 | >3000m | 1141.461 | 0.564148 | 2.598609 | 0.264015 | Tanguticum Maxim. ex Balf. | | |
| 57 | >3000m | 1034.518 | 1.072677 | 3.470326 | 0.357304 | Tanguticum Maxim. ex Balf. | | |
| 83 | >3000m | 619.0801 | 0.540016 | 2.456288 | 0.27133 | Tanguticum Maxim. ex Balf. | | |
| 62 | >3000m | 592.6229 | 0.489937 | 2.280453 | 0.266422 | Tanguticum Maxim. ex Balf. | | |
| 59 | >3000m | 512.2628 | 0.742188 | 2.837823 | 0.312 | Tanguticum Maxim. ex Balf. | | |
| 98 | >3000m | 472.119 | 0.415181 | 2.092006 | 0.254051 | Tanguticum Maxim. ex Balf. | | |
| 72 | >3000m | 429.9244 | 0.439203 | 2.217078 | 0.250381 | Tanguticum Maxim. ex Balf. | | |
| 79 | >3000m | 408.7244 | 0.278214 | 1.792991 | 0.206816 | Tanguticum Maxim. ex Balf. | | |
| 102 | >3000m | 383.9261 | 0.410787 | 2.089461 | 0.250394 | Tanguticum Maxim. ex Balf. | | |
| 95 | >3000m | 370.5165 | 0.482558 | 2.260611 | 0.256973 | Tanguticum Maxim. ex Balf. | | |
| 103 | >3000m | 324.1238 | 0.260316 | 1.689702 | 0.204962 | Tanguticum Maxim. ex Balf. | | |
| 80 | >3000m | 318.1147 | 0.331433 | 1.918667 | 0.223724 | Tanguticum Maxim. ex Balf. | | |
| 71 | >3000m | 311.4923 | 0.411675 | 2.080581 | 0.253935 | Tanguticum Maxim. ex Balf. | | |
| 87 | >3000m | 305.1933 | 0.400472 | 2.077544 | 0.247561 | Tanguticum Maxim. ex Balf. | | |
| 88 | >3000m | 298.3551 | 0.351282 | 1.925939 | 0.238626 | Tanguticum Maxim. ex Balf. | | |
| 70 | >3000m | 278.025 | 0.442147 | 2.250561 | 0.246717 | Tanguticum Maxim. ex Balf. | | |
| 101 | >3000m | 268.2394 | 0.325077 | 1.81886 | 0.235971 | Tanguticum Maxim. ex Balf. | | |
| 84 | >3000m | 265.8944 | 0.221993 | 1.612807 | 0.18781 | Tanguticum Maxim. ex Balf. | | |
| 82 | >3000m | 255.201 | 0.328475 | 1.811805 | 0.23872 | Tanguticum Maxim. ex Balf. | | |
| 55 | >3000m | 245.0448 | 0.165899 | 1.408968 | 0.170895 | Tanguticum Maxim. ex Balf. | | |
| 68 | >3000m | 244.9244 | 0.409565 | 2.359573 | 0.208215 | Tanguticum Maxim. ex Balf. | | |
| 74 | >3000m | 232.0665 | 0.233522 | 1.526464 | 0.216052 | Tanguticum Maxim. ex Balf. | | |
| 113 | >3000m | 227.8894 | 0.364959 | 2.029185 | 0.222786 | Tanguticum Maxim. ex Balf. | | |
| 56 | >3000m | 218.2895 | 0.836996 | 2.917559 | 0.34111 | Tanguticum Maxim. ex Balf. | | |
| 73 | >3000m | 213.0951 | 0.230354 | 1.486199 | 0.217506 | Tanguticum Maxim. ex Balf. | | |
| 69 | >3000m | 211.3089 | 0.440249 | 2.178771 | 0.248327 | Tanguticum Maxim. ex Balf. | | |
| 85 | >3000m | 210.3028 | 0.132474 | 1.273988 | 0.157041 | Tanguticum Maxim. ex Balf. | | |
| 106 | >3000m | 201.2194 | 0.177851 | 1.433489 | 0.178977 | Tanguticum Maxim. ex Balf. | | |
| 99 | >3000m | 197.1809 | 0.197553 | 1.409334 | 0.199057 | Tanguticum Maxim. ex Balf. | | |
| 100 | >3000m | 184.119 | 0.243082 | 1.510415 | 0.220476 | Tanguticum Maxim. ex Balf. | | |
| 75 | >3000m | 173.1548 | 0.141029 | 1.223899 | 0.176281 | Tanguticum Maxim. ex Balf. | | |
| 53 | >3000m | 172.3091 | 0.165817 | 1.307375 | 0.189768 | Tanguticum Maxim. ex Balf. | | |
| 78 | >3000m | 171.0199 | 0.056764 | 0.900699 | 0.099827 | Tanguticum Maxim. ex Balf. | | |
| 81 | >3000m | 170.9311 | 0.340586 | 1.842733 | 0.245426 | Tanguticum Maxim. ex Balf. | | |
| 76 | >3000m | 158.4863 | 0.152887 | 1.224511 | 0.189728 | Tanguticum Maxim. ex Balf. | | |
| 112 | >3000m | 151.2873 | 0.324665 | 1.685703 | 0.249142 | Tanguticum Maxim. ex Balf. | | |
| 111 | >3000m | 142.8997 | 0.294848 | 1.60055 | 0.253942 | Tanguticum Maxim. ex Balf. | | |
| 110 | >3000m | 137.8122 | 0.147169 | 1.250153 | 0.174458 | Tanguticum Maxim. ex Balf. | | |
| 67 | >3000m | 137.7601 | 0.089647 | 1.079129 | 0.12599 | Tanguticum Maxim. ex Balf. | | |
