# Supplementary material for: Application of delayed luminescence measurements for the identification of herbal materials: a step toward rapid quality control
Source: Chin Med. 2019 Oct 28;14:47. doi: 10.1186/s13020-019-0269-2 (PMC6819577; doi:10.1186/s13020-019-0269-2)
Supplement: Supplementary file 2 — Additional file 2: Figure S1. The differences of DL properties between authentic and sulfur fumigation herbal materials. A1) Angelicae dahuricae radix (sliced materials); A2) Angelicae dahuricae radix (sulphur fumigation sample, sliced materials); A3) DL decay curves. Data are plotted as the mean ± SEM. Note that the data are plotted on a log-log scale; A4) Histograms comparing the DL properties between Angelicae dahuricae radix and its sulphur fumigation sample. Mean ± SD, *, p < 0.05; B1) Codonopsis radix (whole materials); B2) Codonopsis radix (sulphur fumigation sample, whole materials); B3) DL decay curves. Data are plotted as the mean ± SEM. Note that the data are plotted on a log-log scale; B4) Histograms comparing the DL properties between Codonopsis radix and its sulphur fumigation sample. Mean ± SD, *, p < 0.05, ns, no significant difference. [file 13020_2019_269_MOESM2_ESM.pdf]

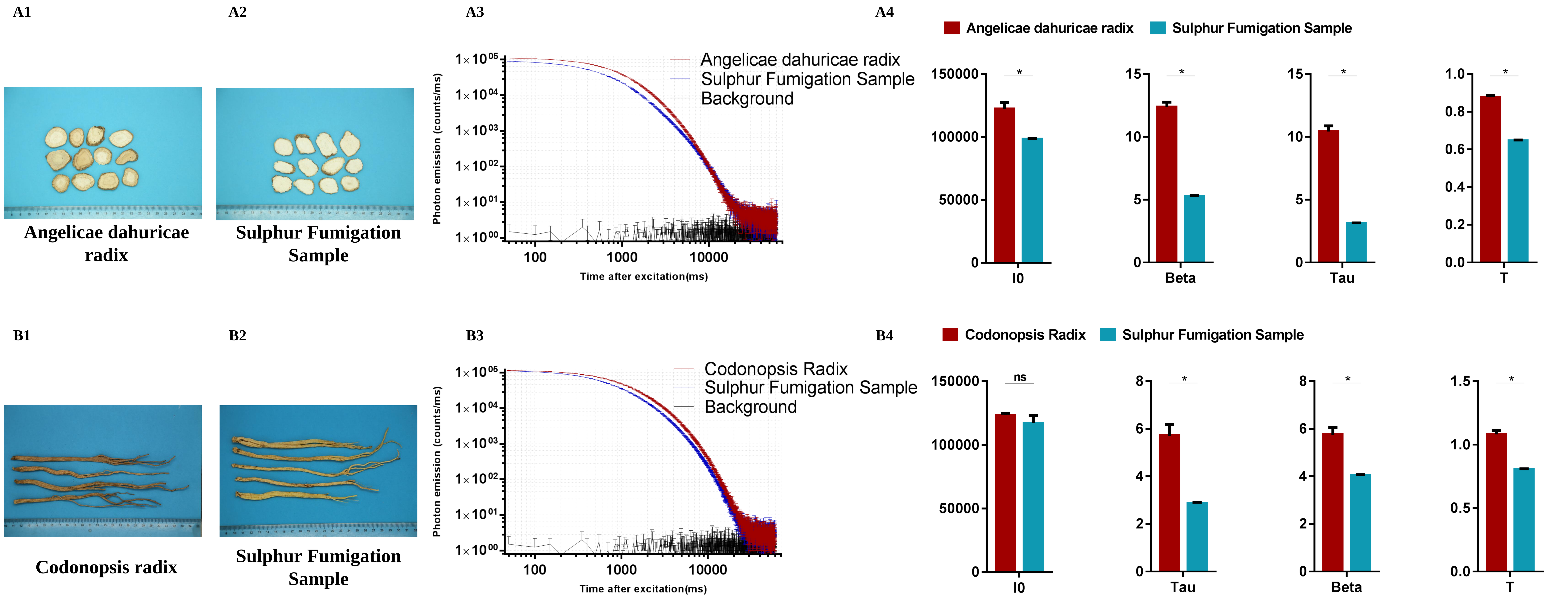

A1
Angelicae dahuricae radix
A2
Sulphur Fumigation Sample
A3
Angelicae dahuricae radix
Sulphur Fumigation Sample
Background
Photon emission (counts/ms)
Time after excitation(ms)
A4
Angelicae dahuricae radix
Sulphur Fumigation Sample
I0
Beta
Tau
T
B1
Codonopsis radix
B2
Sulphur Fumigation Sample
B3
Codonopsis Radix
Sulphur Fumigation Sample
Background
Photon emission (counts/ms)
Time after excitation(ms)
B4
Codonopsis Radix
Sulphur Fumigation Sample
ns
I0
Tau
Beta
T
